# Supplementary material for: Immortalization of human hepatocytes from biliary atresia with CDK4R24C, cyclin D1, and TERT for cytochrome P450 induction testing
Source: Sci Rep. 2020 Oct 15;10:17503. doi: 10.1038/s41598-020-73992-3 (PMC7567112; doi:10.1038/s41598-020-73992-3)
Supplement: Supplementary file 1 — Supplementary legends [file 41598_2020_73992_MOESM1_ESM.docx]

# Supplemental Figures and Tables

## Supplemental Figure 1. Implantation of non-immortalized hepatocytes (Hep2001) into NOD/SCID IL-2 receptor γ -/- (NOG) immunodeficient mice

Hepatocytes (1.5 x 10^7^ cells) were injected into the thigh muscles of NOG mice. The samples were resected at 4 weeks after the implantation and analyzed for histology and immunohistology. Hepatocytes are shown by dashed lines. A. Hematoxylin-eosin (HE) stain. B to D. Immunohistochemistry of the implanted hepatocytes by using the antibodies to vimentin (B), albumin (C: ALB), and cytokeratin 8/18 (D: CK8/18). The hepatocytes are negative for vimentin and positive for albumin and cytokeratin 8/18. The vimentin-positive, albumin-negative, cytokeratin 8/18-negative cells around vimentin-negative hepatocytes are mesenchymal cells in the sample Hep2001.

## Supplemental Figure 2. Immunochemical analysis of HepaMN cells in vitro and in vivo with an antibody to Ki67

**(A).** Immunohistochemistry of HepaMN cells with an antibody to Ki67. (Left) HepaMN cells in culture were harvested with a cell scraper and embedded in an iPGell kit (GenoStaff, Tokyo, Japan). (Right) HepaMN cells were injected into subcutaneous tissue of immunodeficient SCID mice. The HepaMN cell-generated mass was resected for the immunohistochemical analysis. **(B).** Percentage of Ki67-positive cells in HepaMN cells in vitro and in vivo.

## Supplemental Figure 3. Gene induction test for CYP1A1 and CYP1A2 in HepaMN cells

Quantitative RT-PCR analysis of the genes for CYP1A1 (A, C) and CYP1A2 (B, D) was performed on HepaMN cells with exposure to omeprazole (A, B) or β-naphthoflavone (C, D) at the indicated concentration for 24 h.

## Supplemental Figure 4. Cytochrome P450 gene induction test in primary human hepatocytes

**(A).** Quantitative RT-PCR analysis of the genes for CYP1A2 on primary hepatocytes (Bioreclamation IVT, USA, lot #AFJ) with exposure to omeprazole for 24 h at the indicated concentration. mRNA levels were normalized using GAPDH as a housekeeping gene. **(B).** Quantitative RT-PCR analysis of the genes for CYP3A4 on primary hepatocytes (Bioreclamation IVT, USA, lot #AFJ) with exposure to rifampicin for 48 h at the indicated concentration. mRNA levels were normalized using GAPDH as a housekeeping gene. **(C).** Quantitative RT-PCR analysis of the CYP2B6 gene on primary human hepatocytes (Bioreclamation IVT, USA, lot #FOS) with exposure to 500 μM phenobarbital for 48 h. mRNA levels were normalized using GAPDH as a housekeeping gene.

## Supplemental Table 1. Primer pairs and experimental conditions for RT-PCR

## Supplemental Table 2. Liver-associated genes

(A). Developmental markers.

(B). Mature hepatocyte markers.

## Supplemental Table 3. List of immortalized hepatocytes

## Supplemental Table 4. List of liver samples

## Supplemental Table 5. Hepatic differentiation stage of hESCs used for principal component analysis (PCA)

Human embryonic stem cell (hESC) lines SEES1, SEES4, and SEES5 were stably maintained in XF hESC culture medium containing 85% Knockout DMEM, 15% Knockout Serum Replacement XF CTS, 2 mM GlutaMAX-I, 0.1 mM NEAA, Pen-Strep, 50 µg/mL L-ascorbic acid 2-phosphate, 10 ng/mL heregulin-1β (recombinant human NRG-beta 1/HRG-beta 1 EGF domain), 200 ng/mL recombinant human IGF-1 (LONG R3-IGF-1; Sigma-Aldrich), and 20 ng/mL human bFGF (Akutsu et al., Regenerative Therapy; JCI insight). Undifferentiated hESCs were dissociated using dispase and plated on a dish coated with 0.1% human recombinant type I collagen peptide in 90 mm culture dishes. For hepatic differentiation, hESCs were cultured in XF hESC medium without growth factors (XF-KSR(-)) for 1 day and then in XF-KSR medium, which was replaced after 3 days with the XF hESC medium used as the differentiation medium. The differentiation medium was gently changed every 3–4 days until the indicated day.
