# Supplementary material for: Immortalization of human hepatocytes from biliary atresia with CDK4R24C, cyclin D1, and TERT for cytochrome P450 induction testing
Source: Sci Rep. 2020 Oct 15;10:17503. doi: 10.1038/s41598-020-73992-3 (PMC7567112; doi:10.1038/s41598-020-73992-3)
Supplement: Supplementary file 2 — Supplementary information [file 41598_2020_73992_MOESM2_ESM.pdf]

Immortalization of human hepatocytes from biliary atresia with $CDK4^{R24C}$, cyclin D1, and TERT for cytochrome P450 induction testing

Manami Nishiwaki [1], Masashi Toyoda [1, 2], Yoshie Oishi [1], Seiichi Ishida [3], Shin-ichiro Horiuchi [3], Hatsune Makino-Itou [1], Tohru Kimura [4], Shin-ichi Ohno [1], Takashi Ohkura [1], Shin Enosawa [5], Hidenori Akutsu [1], Atsuko Nakazawa [1, 6], Mureo Kasahara [7], Tohru Kiyono [8*], and Akihiro Umezawa [1*]

[1] Center for Regenerative Medicine, National Center for Child Health and Development Research Institute, Tokyo, 157-8535, Japan
[2] Research team for Geriatric Medicine (Vascular Medicine), Tokyo Metropolitan Institute of Gerontology, Tokyo, 173-0015, Japan
[3] Division of Pharmacology, National Institute of Health Sciences, Kanagawa, 210-9501, Japan
[4] Laboratory of Stem Cell Biology, Department of Biosciences, Kitasato University School of Science, Kanagawa 252-0373, Japan
[5] Division for Advanced Medical Sciences, National Center for Child Health and Development, Tokyo, 157-8535, Japan
[6] Saitama Children's Medical Center, Saitama, 330-8777, Japan
[7] Organ Transplantation Center, National Center for Child Health and Development, Tokyo, 157-8535, Japan
[8] Project for Prevention of HPV-related Cancer, Exploratory Oncology Research and Clinical Trial Center, National Cancer Center, Chiba, 277-8577, Japan

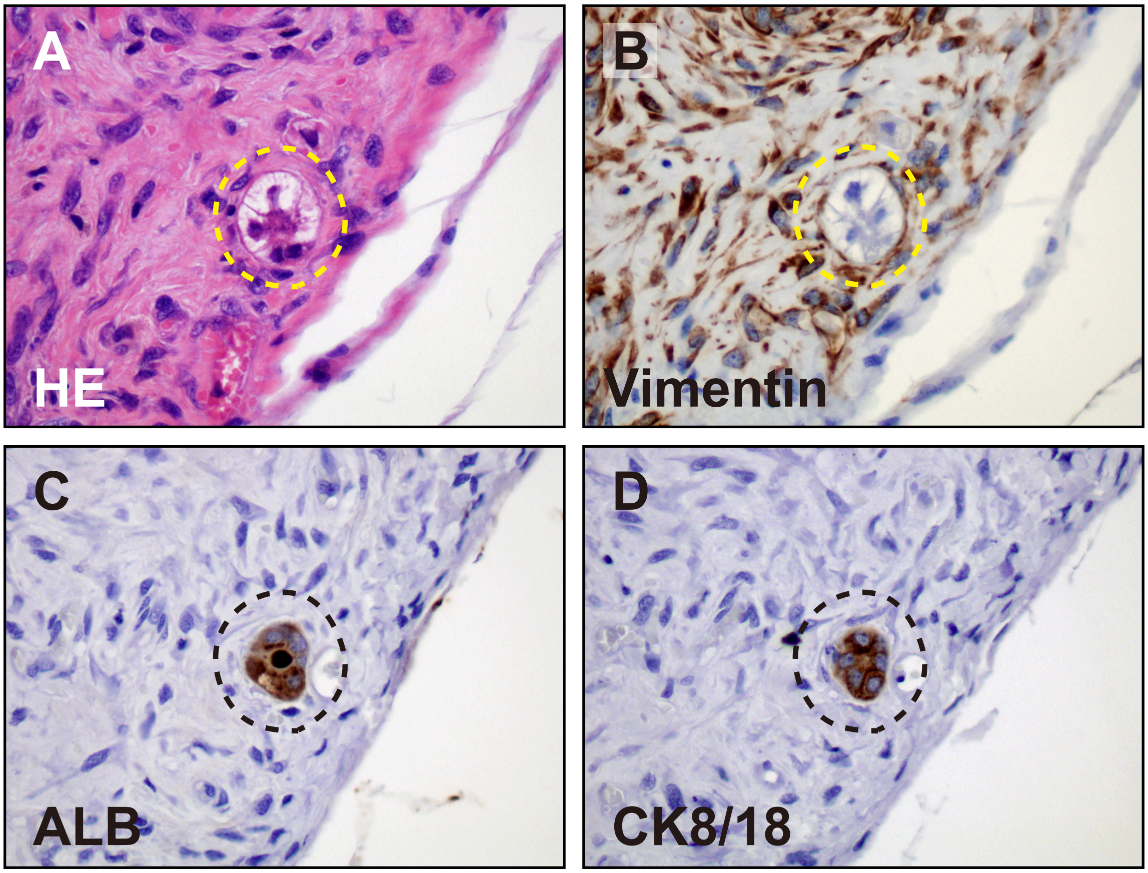

Supplemental Figure 1

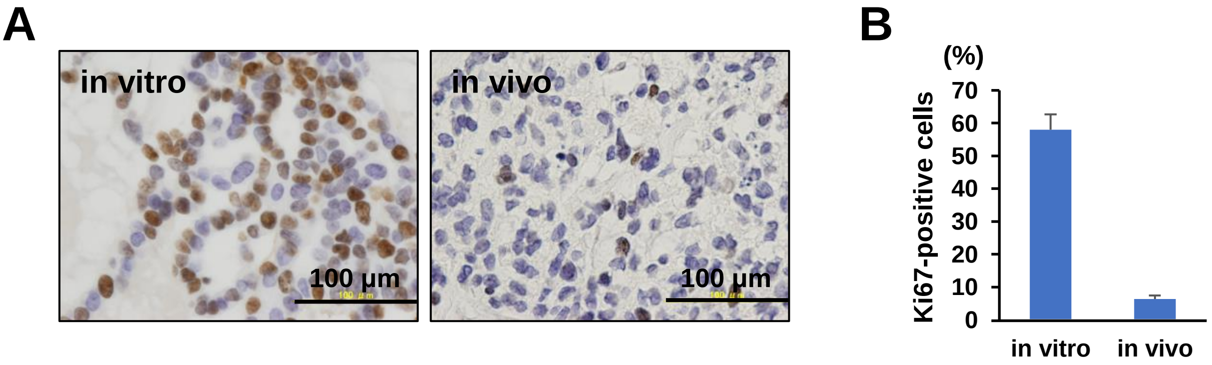

Supplemental Figure 2

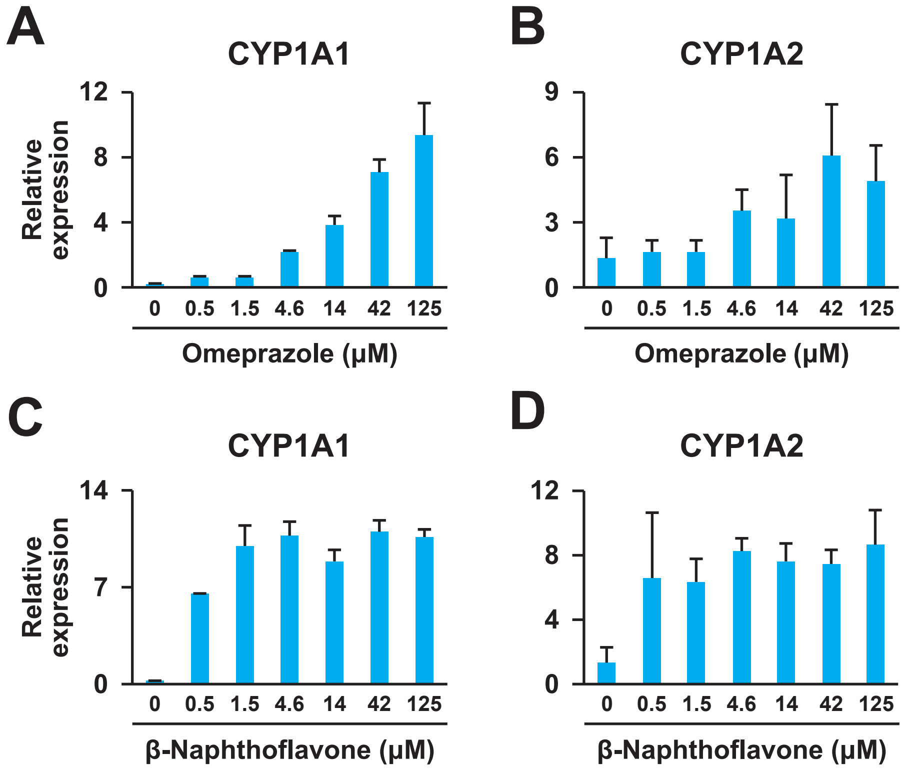

Supplemental Figure 3

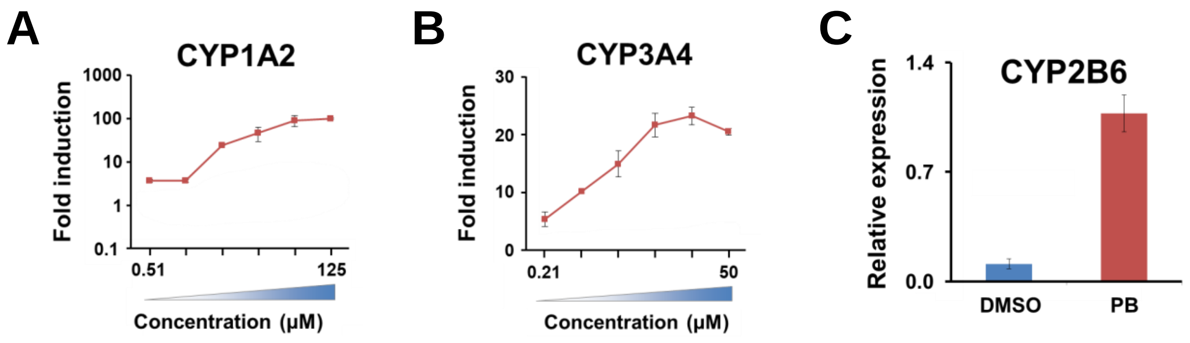

Supplemental Figure 4

Original images in Figure 1I

CDK4

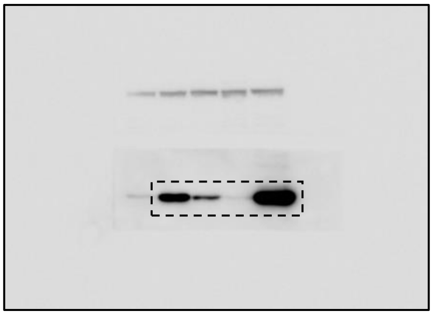

Cyclin D1

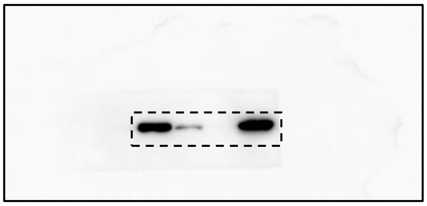

Vinculin

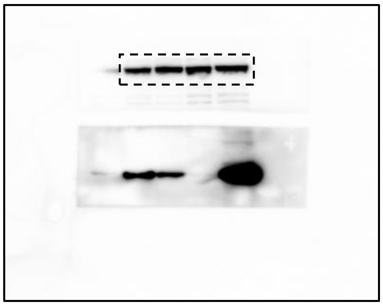

Original image in Figure 1J

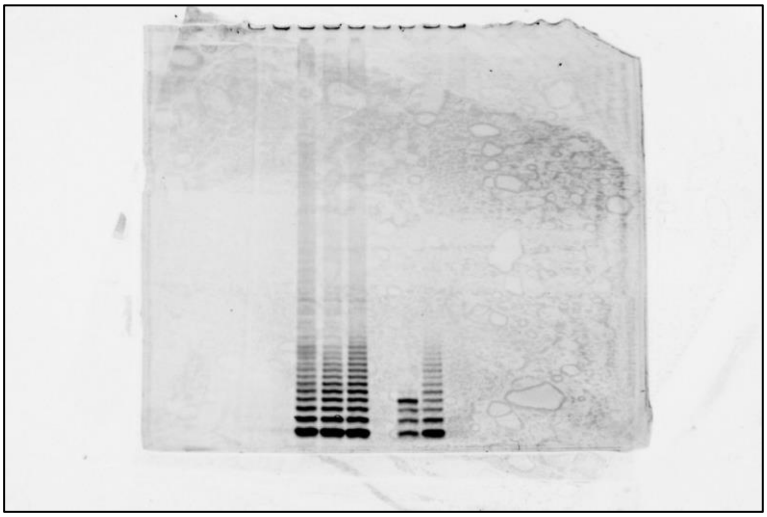

Supplemental Table 1. Primer pairs and experimental conditions for RT-PCR

Gene product	Forward and reverse primers (5'–3')	Expected product size
AFP	AGCTTGGTGGTGGATGAAAC	248
	CCCTCTTCAGCAAAGCAGAC	
ALB	TGGCACAATGAAGTGGGTAA	166
	CTGAGCAAAGGCAATCAACA	
CYP1A2	CAATCAGGTGGTGGTGTCAG	245
	GCTCCTGGACTGTTTTCTGC	
CYP2B6	TCCTTTCTGAGGTTCCGAGA	416
	TCCCGAAGTCCCTCATAGTG	
CYP3A4	CAAGACCCCTTTGTGGAAAA	187
	CGAGGCGACTTTCTTTCATC	
AAT	GGGAAACTACAGCACCTGGA	175
	CCCCATTGCTGAAGACCTTA	
TDO2	GGGAACTACCTGCATTTGGA	222
	GTGCATCCGAGAAACAACCT	
OTC	ACCTTCAGGCAGCTACTCCA	192
	GCCGCTTTTTCTTCTCCTCT	
ARG	GGCTGGTCTGCTTGAGAAAC	240
	ATTGCCAAACTGTGGTCTCC	
UBIQUITIN	GGAGCCGAGTGACACCATTG	346
	CAGGGTACGACCATCTTCCAG	

Supplemental Table 2. Liver-associated genes

A. Developmental markers

ProbeName	GeneSymbol	GeneName	Description
A_23_P204395	AACS	acetoacetyl-CoA synthetase	acetoacetyl-CoA synthetase (AACS)
A_33_P3403392	AACS	acetoacetyl-CoA synthetase	acetoacetyl-CoA synthetase (AACS)
A_23_P24515	ACAT1	acetyl-CoA acetyltransferase 1	acetyl-CoA acetyltransferase 1 (ACAT1)
A_24_P203678	ACAT1	acetyl-CoA acetyltransferase 1	acetyl-CoA acetyltransferase 1 (ACAT1)
A_33_P3253975	ACAT1	acetyl-CoA acetyltransferase 1	acetyl-CoA acetyltransferase 1 (ACAT1)
A_23_P200404	AK2	adenylate kinase 2	adenylate kinase 2 (AK2)
A_24_P179903	AK2	adenylate kinase 2	adenylate kinase 2 (AK2)
A_24_P500891	AK2	adenylate kinase 2	adenylate kinase 2 (AK2)
A_33_P3392580	AK2	adenylate kinase 2	adenylate kinase 2 (AK2)
A_24_P73577	ALDH1A2	aldehyde dehydrogenase 1 family, member A2	aldehyde dehydrogenase 1 family, member A2 (ALDH1A2)
A_32_P18440	ARID5B	AT rich interactive domain 5B (MRF1-like)	AT rich interactive domain 5B (MRF1-like) (ARID5B)
A_33_P3324980	ARID5B	AT rich interactive domain 5B (MRF1-like)	AT rich interactive domain 5B (MRF1-like) (ARID5B)
A_23_P145694	ASNS	asparagine synthetase (glutamine-hydrolyzing)	asparagine synthetase (glutamine-hydrolyzing) (ASNS)
A_23_P31921	ASS1	argininosuccinate synthase 1	argininosuccinate synthase 1 (ASS1)
A_33_P3234580	ASS1	argininosuccinate synthase 1	argininosuccinate synthase 1 (ASS1)
A_23_P143987	ATG7	autophagy related 7	autophagy related 7 (ATG7)
A_24_P944827	ATG7	autophagy related 7	autophagy related 7 (ATG7)
A_33_P3252359	BDH1	3-hydroxybutyrate dehydrogenase, type 1	3-hydroxybutyrate dehydrogenase, type 1 (BDH1)
A_33_P3211138	CADM1	cell adhesion molecule 1	cell adhesion molecule 1 (CADM1)
A_33_P3421913	CADM1	cell adhesion molecule 1	cell adhesion molecule 1 (CADM1)
A_33_P3745146	CADM1	cell adhesion molecule 1	cell adhesion molecule 1 (CADM1)
A_32_P404549	CCDC39	coiled-coil domain containing 39	coiled-coil domain containing 39 (CCDC39)
A_24_P160680	CCDC40	coiled-coil domain containing 40	coiled-coil domain containing 40 (CCDC40)
A_33_P3302518	CCDC40	coiled-coil domain containing 40	coiled-coil domain containing 40 (CCDC40)
A_33_P3253807	CEBPG	CCAAT/enhancer binding protein (C/EBP), gamma	CCAAT/enhancer binding protein (C/EBP), gamma (CEBPG)
A_21_P0011496	CES1	carboxylesterase 1	carboxylesterase 1 (CES1)
A_23_P206733	CES1	carboxylesterase 1	carboxylesterase 1 (CES1)
A_33_P3241269	CES1	carboxylesterase 1	carboxylesterase 1 (CES1)
A_33_P3389704	CES1	carboxylesterase 1	carboxylesterase 1 (CES1)
A_23_P214969	CITED2	Cbp/p300-interacting transactivator, with Glu/Asp-rich carboxy-terminal doma	Cbp/p300-interacting transactivator, with Glu/Asp-rich carboxy-terminal domain, 2 (CITED2)
A_33_P3213374	CITED2	Cbp/p300-interacting transactivator, with Glu/Asp-rich carboxy-terminal doma	Cbp/p300-interacting transactivator, with Glu/Asp-rich carboxy-terminal domain, 2 (CITED2)
A_33_P3327673	COBL	cordon-bleu WH2 repeat protein	cordon-bleu WH2 repeat protein (COBL)
A_33_P3725227	COBL	cordon-bleu WH2 repeat protein	cordon-bleu WH2 repeat protein (COBL)
A_33_P3242798	CPS1	carbamoyl-phosphate synthase 1, mitochondrial	carbamoyl-phosphate synthase 1, mitochondrial (CPS1)
A_23_P29495	CTNNB1	catenin (cadherin-associated protein), beta 1, 88kDa	catenin (cadherin-associated protein), beta 1, 88kDa (CTNNB1)
A_33_P3421695	CTNNB1	catenin (cadherin-associated protein), beta 1, 88kDa	catenin (cadherin-associated protein), beta 1, 88kDa (CTNNB1)
A_23_P163402	CYP1A1	cytochrome P450, family 1, subfamily A, polypeptide 1	cytochrome P450, family 1, subfamily A, polypeptide 1 (CYP1A1)
A_23_P130753	DBP	D site of albumin promoter (albumin D-box) binding protein	D site of albumin promoter (albumin D-box) binding protein (DBP)
A_23_P54612	DNAAF1	dynein, axonemal, assembly factor 1	dynein, axonemal, assembly factor 1 (DNAAF1)

A_33_P3368388	DNAAF1	dynein, axonemal, assembly factor 1	dynein, axonemal, assembly factor 1 (DNAAF1)
A_32_P210202	E2F7	E2F transcription factor 7	E2F transcription factor 7 (E2F7)
A_33_P3318661	E2F7	E2F transcription factor 7	E2F transcription factor 7 (E2F7)
A_23_P35871	E2F8	E2F transcription factor 8	E2F transcription factor 8 (E2F8)
A_23_P215790	EGFR	epidermal growth factor receptor	epidermal growth factor receptor (EGFR)
A_33_P3351944	EGFR	epidermal growth factor receptor	epidermal growth factor receptor (EGFR)
A_33_P3351955	EGFR	epidermal growth factor receptor	epidermal growth factor receptor (EGFR)
A_24_P397150	GAK	cyclin G associated kinase	cyclin G associated kinase (GAK)
A_33_P3254013	GAK	cyclin G associated kinase	cyclin G associated kinase (GAK)
A_23_P429184	GNPNAT1	glucosamine-phosphate N-acetyltransferase 1	glucosamine-phosphate N-acetyltransferase 1 (GNPNAT1)
A_33_P3276713	HGF	hepatocyte growth factor (hepapoietin A; scatter factor)	hepatocyte growth factor (hepapoietin A; scatter factor) (HGF)
A_33_P3276718	HGF	hepatocyte growth factor (hepapoietin A; scatter factor)	hepatocyte growth factor (hepapoietin A; scatter factor) (HGF)
A_23_P47034	HHEX	hematopoietically expressed homeobox	hematopoietically expressed homeobox (HHEX)
A_23_P126266	HLX	H2.0-like homeobox	H2.0-like homeobox (HLX)
A_24_P63522	HMGCS1	3-hydroxy-3-methylglutaryl-CoA synthase 1 (soluble)	3-hydroxy-3-methylglutaryl-CoA synthase 1 (soluble) (HMGCS1)
A_33_P3299119	HNF1A	HNF1 homeobox A	HNF1 homeobox A (HNF1A)
A_33_P3299122	HNF1A	HNF1 homeobox A	HNF1 homeobox A (HNF1A)
A_33_P3328312	HNF1A	HNF1 homeobox A	HNF1 homeobox A (HNF1A)
A_23_P406782	HPN	hepsin	hepsin (HPN)
A_24_P128524	ICMT	isoprenylcysteine carboxyl methyltransferase	isoprenylcysteine carboxyl methyltransferase (ICMT)
A_33_P3381022	ICMT	isoprenylcysteine carboxyl methyltransferase	isoprenylcysteine carboxyl methyltransferase (ICMT)
A_23_P48339	IFT88	intraflagellar transport 88	intraflagellar transport 88 (IFT88)
A_32_P178800	ITGA2	integrin, alpha 2 (CD49B, alpha 2 subunit of VLA-2 receptor)	integrin, alpha 2 (CD49B, alpha 2 subunit of VLA-2 receptor) (ITGA2)
A_23_P142389	LSR	lipolysis stimulated lipoprotein receptor	lipolysis stimulated lipoprotein receptor (LSR)
A_33_P3214466	MESP1	mesoderm posterior basic helix-loop-helix transcription factor 1	mesoderm posterior basic helix-loop-helix transcription factor 1 (MESP1)
A_23_P54556	MKL2	MKL/myocardin-like 2	MKL/myocardin-like 2 (MKL2)
A_24_P375205	MKL2	MKL/myocardin-like 2	cDNA FLJ36258 fis, clone THYMU2002450
A_21_P0012236	NF1	neurofibromin 1	neurofibromin 1 (NF1)
A_24_P1919	NF1	neurofibromin 1	neurofibromin 1 (NF1)
A_24_P917026	NF1	neurofibromin 1	neurofibromin 1 (NF1)
A_33_P3240348	NF1	neurofibromin 1	neurofibromin 1 (NF1)
A_33_P3381490	NKX2-8	NK2 homeobox 8	NK2 homeobox 8 (NKX2-8)
A_33_P3228072	NPHP3	nephronophthisis 3 (adolescent)	nephronophthisis 3 (adolescent) (NPHP3)
A_33_P3228102	NPHP3	nephronophthisis 3 (adolescent)	nephronophthisis 3 (adolescent) (NPHP3)
A_32_P142440	PCSK9	proprotein convertase subtilisin/kexin type 9	proprotein convertase subtilisin/kexin type 9 (PCSK9)
A_23_P502371	PHF2	PHD finger protein 2	PHD finger protein 2 (PHF2)
A_24_P106112	PKD2	polycystic kidney disease 2 (autosomal dominant)	polycystic kidney disease 2 (autosomal dominant) (PKD2)
A_24_P396197	PRKCSH	protein kinase C substrate 80K-H	protein kinase C substrate 80K-H (PRKCSH)
A_24_P88266	PROX1	prospero homeobox 1	prospero homeobox 1 (PROX1)
A_24_P191847	PTCD2	pentatricopeptide repeat domain 2	pentatricopeptide repeat domain 2 (PTCD2)
A_33_P3371819	PTCD2	pentatricopeptide repeat domain 2	pentatricopeptide repeat domain 2 (PTCD2)
A_23_P207842	RARA	retinoic acid receptor, alpha	retinoic acid receptor, alpha (RARA)

A_32_P5251	RARA	retinoic acid receptor, alpha	retinoic acid receptor, alpha (RARA)
A_23_P9056	RB1CC1	RB1-inducible coiled-coil 1	RB1-inducible coiled-coil 1 (RB1CC1)
A_23_P104689	RELA	v-rel avian reticuloendotheliosis viral oncogene homolog A	v-rel avian reticuloendotheliosis viral oncogene homolog A (RELA)
A_33_P3209433	RELA	v-rel avian reticuloendotheliosis viral oncogene homolog A	v-rel avian reticuloendotheliosis viral oncogene homolog A (RELA)
A_33_P3274069	RHBDD3	rhomboid domain containing 3	rhomboid domain containing 3 (RHBDD3)
A_33_P3330731	RHBDD3	rhomboid domain containing 3	rhomboid domain containing 3 (RHBDD3)
A_33_P3302861	RPGRIP1L	RPGRIP1-like	RPGRIP1-like (RPGRIP1L)
A_24_P34155	RUNX1	runt-related transcription factor 1	runt-related transcription factor 1 (RUNX1)
A_24_P96403	RUNX1	runt-related transcription factor 1	runt-related transcription factor 1 (RUNX1)
A_33_P3211804	RUNX1	runt-related transcription factor 1	runt-related transcription factor 1 (RUNX1)
A_33_P3211809	RUNX1	runt-related transcription factor 1	runt-related transcription factor 1 (RUNX1)
A_33_P3211818	RUNX1	runt-related transcription factor 1	runt-related transcription factor 1 (RUNX1)
A_23_P214977	SEC63	SEC63 homolog (S. cerevisiae)	SEC63 homolog (S. cerevisiae) (SEC63)
A_24_P123720	SEC63	SEC63 homolog (S. cerevisiae)	SEC63 homolog (S. cerevisiae) (SEC63)
A_23_P150768	SLCO2B1	solute carrier organic anion transporter family, member 2B1	solute carrier organic anion transporter family, member 2B1 (SLCO2B1)
A_23_P48936	SMAD3	SMAD family member 3	SMAD family member 3 (SMAD3)
A_23_P134176	SOD2	superoxide dismutase 2, mitochondrial	superoxide dismutase 2, mitochondrial (SOD2)
A_33_P3380867	STAT5B	signal transducer and activator of transcription 5B	signal transducer and activator of transcription 5B (STAT5B)
A_23_P200780	TGFBR3	transforming growth factor, beta receptor III	transforming growth factor, beta receptor III (TGFBR3)
A_24_P157926	TNFAIP3	tumor necrosis factor, alpha-induced protein 3	tumor necrosis factor, alpha-induced protein 3 (TNFAIP3)
A_23_P47704	UCP2	uncoupling protein 2 (mitochondrial, proton carrier)	uncoupling protein 2 (mitochondrial, proton carrier) (UCP2)
A_23_P327910	ZIC3	Zic family member 3	Zic family member 3 (ZIC3)
A_33_P3250861	ZIC3	Zic family member 3	Zic family member 3 (ZIC3)

B. Mature hepatocyte markers

ProbeName	GeneSymbol	GeneName	Description
A_23_P257834	ALB	albumin	albumin (ALB)
A_33_P3242798	CPS1	carbamoyl-phosphate synthase 1, mitochondrial	carbamoyl-phosphate synthase 1, mitochondrial (CPS1)
A_32_P73821	CSDE1	cold shock domain containing E1, RNA-binding	cold shock domain containing E1, RNA-binding (CSDE1)
A_23_P129169	CYP11A1	cytochrome P450, family 11, subfamily A, polypeptide 1	cytochrome P450, family 11, subfamily A, polypeptide 1 (CYP11A1)
A_24_P329424	CYP11B1	cytochrome P450, family 11, subfamily B, polypeptide 1	cytochrome P450, family 11, subfamily B, polypeptide 1 (CYP11B1)
A_23_P215997	CYP11B2	cytochrome P450, family 11, subfamily B, polypeptide 2	cytochrome P450, family 11, subfamily B, polypeptide 2 (CYP11B2)
A_33_P3376478	CYP17A1	cytochrome P450, family 17, subfamily A, polypeptide 1	cytochrome P450, family 17, subfamily A, polypeptide 1 (CYP17A1)
A_24_P920646	CYP19A1	cytochrome P450, family 19, subfamily A, polypeptide 1	cytochrome P450, family 19, subfamily A, polypeptide 1 (CYP19A1)
A_32_P86289	CYP19A1	cytochrome P450, family 19, subfamily A, polypeptide 1	cytochrome P450, family 19, subfamily A, polypeptide 1 (CYP19A1)
A_23_P37410	CYP19A1	cytochrome P450, family 19, subfamily A, polypeptide 1	cytochrome P450, family 19, subfamily A, polypeptide 1 (CYP19A1)
A_33_P3351371	CYP19A1	cytochrome P450, family 19, subfamily A, polypeptide 1	cytochrome P450, family 19, subfamily A, polypeptide 1 (CYP19A1)
A_23_P163402	CYP1A1	cytochrome P450, family 1, subfamily A, polypeptide 1	cytochrome P450, family 1, subfamily A, polypeptide 1 (CYP1A1)
A_33_P3253747	CYP1A2	cytochrome P450, family 1, subfamily A, polypeptide 2	cytochrome P450, family 1, subfamily A, polypeptide 2 (CYP1A2)
A_23_P209625	CYP1B1	cytochrome P450, family 1, subfamily B, polypeptide 1	cytochrome P450, family 1, subfamily B, polypeptide 1 (CYP1B1)
A_33_P3290343	CYP1B1	cytochrome P450, family 1, subfamily B, polypeptide 1	cytochrome P450, family 1, subfamily B, polypeptide 1 (CYP1B1)
A_22_P00006287	CYP1B1-AS1	CYP1B1 antisense RNA 1	CYP1B1 antisense RNA 1 (CYP1B1-AS1)
A_19_P00807643	CYP1B1-AS1	CYP1B1 antisense RNA 1	CYP1B1 antisense RNA 1 (CYP1B1-AS1)

A_23_P56894	CYP20A1	cytochrome P450, family 20, subfamily A, polypeptide 1	cytochrome P450, family 20, subfamily A, polypeptide 1 (CYP20A1)
A_33_P3371175	CYP20A1	cytochrome P450, family 20, subfamily A, polypeptide 1	cytochrome P450, family 20, subfamily A, polypeptide 1 (CYP20A1)
A_23_P257478	CYP21A2	cytochrome P450, family 21, subfamily A, polypeptide 2	cytochrome P450, family 21, subfamily A, polypeptide 2 (CYP21A2)
A_33_P3411279	CYP21A2	cytochrome P450, family 21, subfamily A, polypeptide 2	cytochrome P450, family 21, subfamily A, polypeptide 2 (CYP21A2)
A_23_P28815	CYP24A1	cytochrome P450, family 24, subfamily A, polypeptide 1	cytochrome P450, family 24, subfamily A, polypeptide 1 (CYP24A1)
A_33_P3369401	CYP24A1	cytochrome P450, family 24, subfamily A, polypeptide 1	cytochrome P450, family 24, subfamily A, polypeptide 1 (CYP24A1)
A_23_P138655	CYP26A1	cytochrome P450, family 26, subfamily A, polypeptide 1	cytochrome P450, family 26, subfamily A, polypeptide 1 (CYP26A1)
A_23_P210109	CYP26B1	cytochrome P450, family 26, subfamily B, polypeptide 1	cytochrome P450, family 26, subfamily B, polypeptide 1 (CYP26B1)
A_33_P3361422	CYP27A1	cytochrome P450, family 27, subfamily A, polypeptide 1	cytochrome P450, family 27, subfamily A, polypeptide 1 (CYP27A1)
A_23_P36397	CYP27B1	cytochrome P450, family 27, subfamily B, polypeptide 1	cytochrome P450, family 27, subfamily B, polypeptide 1 (CYP27B1)
A_23_P55779	CYP2A13	cytochrome P450, family 2, subfamily A, polypeptide 13	cytochrome P450, family 2, subfamily A, polypeptide 13 (CYP2A13)
A_23_P27528	CYP2A7	cytochrome P450, family 2, subfamily A, polypeptide 7	cytochrome P450, family 2, subfamily A, polypeptide 7 (CYP2A7)
A_24_P339514	CYP2B6	cytochrome P450, family 2, subfamily B, polypeptide 6	cytochrome P450, family 2, subfamily B, polypeptide 6 (CYP2B6)
A_23_P208373	CYP2B6	cytochrome P450, family 2, subfamily B, polypeptide 6	cytochrome P450, family 2, subfamily B, polypeptide 6 (CYP2B6)
A_23_P52480	CYP2C18	cytochrome P450, family 2, subfamily C, polypeptide 18	cytochrome P450, family 2, subfamily C, polypeptide 18 (CYP2C18)
A_33_P3326075	CYP2C19	cytochrome P450, family 2, subfamily C, polypeptide 19	cytochrome P450, family 2, subfamily C, polypeptide 19 (CYP2C19)
A_23_P161368	CYP2C8	cytochrome P450, family 2, subfamily C, polypeptide 8	cytochrome P450, family 2, subfamily C, polypeptide 8 (CYP2C8)
A_23_P12767	CYP2C9	cytochrome P450, family 2, subfamily C, polypeptide 9	cytochrome P450, family 2, subfamily C, polypeptide 9 (CYP2C9)
A_23_P155123	CYP2D6	cytochrome P450, family 2, subfamily D, polypeptide 6	cytochrome P450, family 2, subfamily D, polypeptide 6 (CYP2D6)
A_23_P143734	CYP2D6	cytochrome P450, family 2, subfamily D, polypeptide 6	cytochrome P450, family 2, subfamily D, polypeptide 6 (CYP2D6)
A_24_P394940	CYP2E1	cytochrome P450, family 2, subfamily E, polypeptide 1	cytochrome P450, family 2, subfamily E, polypeptide 1 (CYP2E1)
A_23_P89981	CYP2F1	cytochrome P450, family 2, subfamily F, polypeptide 1	cytochrome P450, family 2, subfamily F, polypeptide 1 (CYP2F1)
A_23_P103486	CYP2J2	cytochrome P450, family 2, subfamily J, polypeptide 2	cytochrome P450, family 2, subfamily J, polypeptide 2 (CYP2J2)
A_23_P202860	CYP2R1	cytochrome P450, family 2, subfamily R, polypeptide 1	cytochrome P450, family 2, subfamily R, polypeptide 1 (CYP2R1)
A_21_P0014273	CYP2R1	cytochrome P450, family 2, subfamily R, polypeptide 1	cytochrome P450, family 2, subfamily R, polypeptide 1 (CYP2R1)
A_23_P101374	CYP2S1	cytochrome P450, family 2, subfamily S, polypeptide 1	cytochrome P450, family 2, subfamily S, polypeptide 1
A_33_P3348782	CYP2S1	cytochrome P450, family 2, subfamily S, polypeptide 1	cytochrome P450, family 2, subfamily S, polypeptide 1
A_33_P3252605	CYP2U1	cytochrome P450, family 2, subfamily U, polypeptide 1	cytochrome P450, family 2, subfamily U, polypeptide 1 (CYP2U1)
A_33_P3252612	CYP2W1	cytochrome P450, family 2, subfamily W, polypeptide 1	cytochrome P450, family 2, subfamily W, polypeptide 1 (CYP2W1)
A_23_P133712	CYP39A1	cytochrome P450, family 39, subfamily A, polypeptide 1	cytochrome P450, family 39, subfamily A, polypeptide 1 (CYP39A1)
A_33_P3251342	CYP3A4	cytochrome P450, family 3, subfamily A, polypeptide 4	cytochrome P450, family 3, subfamily A, polypeptide 4 (CYP3A4)
A_23_P215828	CYP3A43	cytochrome P450, family 3, subfamily A, polypeptide 43	cytochrome P450, family 3, subfamily A, polypeptide 43 (CYP3A43)
A_23_P8801	CYP3A5	cytochrome P450, family 3, subfamily A, polypeptide 5	cytochrome P450, family 3, subfamily A, polypeptide 5 (CYP3A5)
A_33_P3249746	CYP3A5	cytochrome P450, family 3, subfamily A, polypeptide 5	cytochrome P450, family 3, subfamily A, polypeptide 5 (CYP3A5)
A_23_P358917	CYP3A7	cytochrome P450, family 3, subfamily A, polypeptide 7	cytochrome P450, family 3, subfamily A, polypeptide 7 (CYP3A7)
A_33_P3318117	CYP3A7	cytochrome P450, family 3, subfamily A, polypeptide 7	cytochrome P450, family 3, subfamily A, polypeptide 7 (CYP3A7)
A_23_P48784	CYP46A1	cytochrome P450, family 46, subfamily A, polypeptide 1	cytochrome P450, family 46, subfamily A, polypeptide 1
A_33_P3337604	CYP46A1	cytochrome P450, family 46, subfamily A, polypeptide 1	cytochrome P450, family 46, subfamily A, polypeptide 1
A_24_P191013	CYP4A11	cytochrome P450, family 4, subfamily A, polypeptide 11	cytochrome P450, family 4, subfamily F, polypeptide 11 (CYP4F11)
A_33_P3303474	CYP4A11	cytochrome P450, family 4, subfamily A, polypeptide 11	cytochrome P450, family 4, subfamily F, polypeptide 11 (CYP4F11)
A_23_P114713	CYP4B1	cytochrome P450, family 4, subfamily B, polypeptide 1	cytochrome P450, family 4, subfamily B, polypeptide 1 (CYP4B1)
A_21_P0000967	CYP4B1	cytochrome P450, family 4, subfamily B, polypeptide 1	cytochrome P450, family 4, subfamily B, polypeptide 1

A_24_P42693	CYP4F11	cytochrome P450, family 4, subfamily F, polypeptide 11	cytochrome P450, family 4, subfamily F, polypeptide 11 (CYP4F11)
A_23_P39315	CYP4F11	cytochrome P450, family 4, subfamily F, polypeptide 11	cytochrome P450, family 4, subfamily F, polypeptide 11 (CYP4F11)
A_23_P108280	CYP4F12	cytochrome P450, family 4, subfamily F, polypeptide 12	cytochrome P450, family 4, subfamily F, polypeptide 12 (CYP4F12)
A_23_P50710	CYP4F2	cytochrome P450, family 4, subfamily F, polypeptide 2	cytochrome P450, family 4, subfamily F, polypeptide 2 (CYP4F2)
A_33_P3359017	CYP4F2	cytochrome P450, family 4, subfamily F, polypeptide 2	cytochrome P450, family 4, subfamily F, polypeptide 2 (CYP4F2)
A_24_P331150	CYP4F22	cytochrome P450, family 4, subfamily F, polypeptide 22	cytochrome P450, family 4, subfamily F, polypeptide 22 (CYP4F22)
A_33_P3294277	CYP4F3	cytochrome P450, family 4, subfamily F, polypeptide 3	cytochrome P450, family 4, subfamily F, polypeptide 3 (CYP4F3)
A_23_P39881	CYP4F30P	cytochrome P450, family 4, subfamily F, polypeptide 30, pseudogene	cytochrome P450, family 4, subfamily F, polypeptide 30, pseudogene (CYP4F30P)
A_24_P28811	CYP4F62P	cytochrome P450, family 4, subfamily F, polypeptide 62, pseudogene	cytochrome P450, family 4, subfamily F, polypeptide 62, pseudogene (CYP4F62P)
A_23_P131060	CYP4F8	cytochrome P450, family 4, subfamily F, polypeptide 8	cytochrome P450, family 4, subfamily F, polypeptide 8 (CYP4F8)
A_24_P945228	CYP4V2	cytochrome P450, family 4, subfamily V, polypeptide 2	cytochrome P450, family 4, subfamily V, polypeptide 2 (CYP4V2)
A_24_P293530	CYP4X1	cytochrome P450, family 4, subfamily X, polypeptide 1	cytochrome P450, family 4, subfamily X, polypeptide 1 (CYP4X1)
A_23_P103971	CYP4Z1	cytochrome P450, family 4, subfamily Z, polypeptide 1	cytochrome P450, family 4, subfamily Z, polypeptide 1 (CYP4Z1)
A_33_P3279880	CYP4Z1	cytochrome P450, family 4, subfamily Z, polypeptide 1	cytochrome P450, family 4, subfamily Z, polypeptide 1 (CYP4Z1)
A_21_P0010756	CYP4Z1	cytochrome P450, family 4, subfamily Z, polypeptide 1	cytochrome P450, family 4, subfamily Z, polypeptide 1 (CYP4Z1)
A_24_P145529	CYP4Z2P	cytochrome P450, family 4, subfamily Z, polypeptide 2, pseudogene	cDNA FLJ40054 fis, clone TBAES2000315
A_21_P0010757	CYP4Z2P	cytochrome P450, family 4, subfamily Z, polypeptide 2, pseudogene	cytochrome P450 (CYP4Z2P)
A_24_P130041	CYP51A1	cytochrome P450, family 51, subfamily A, polypeptide 1	cytochrome P450, family 51, subfamily A, polypeptide 1 (CYP51A1)
A_23_P146198	CYP7A1	cytochrome P450, family 7, subfamily A, polypeptide 1	cytochrome P450, family 7, subfamily A, polypeptide 1 (CYP7A1)
A_23_P169092	CYP7B1	cytochrome P450, family 7, subfamily B, polypeptide 1	cytochrome P450, family 7, subfamily B, polypeptide 1 (CYP7B1)
A_24_P208704	CYP8B1	cytochrome P450, family 8, subfamily B, polypeptide 1	cytochrome P450, family 8, subfamily B, polypeptide 1 (CYP8B1)
A_33_P3733417	DRD2	dopamine receptor D2	dopamine receptor D2 (DRD2)
A_32_P210202	E2F7	E2F transcription factor 7	E2F transcription factor 7 (E2F7)
A_33_P3318661	E2F7	E2F transcription factor 7	E2F transcription factor 7 (E2F7)
A_23_P35871	E2F8	E2F transcription factor 8	E2F transcription factor 8 (E2F8)
A_23_P144843	ESM1	endothelial cell-specific molecule 1	endothelial cell-specific molecule 1 (ESM1)
A_23_P160742	GLMN	glomulin, FKBP associated protein	glomulin, FKBP associated protein (GLMN)
A_23_P47034	HHEX	hematopoietically expressed homeobox	hematopoietically expressed homeobox (HHEX)
A_23_P406782	HPN	hepsin	hepsin (HPN)
A_32_P178800	ITGA2	integrin, alpha 2 (CD49B, alpha 2 subunit of VLA-2 receptor)	integrin, alpha 2 (CD49B, alpha 2 subunit of VLA-2 receptor) (ITGA2)
A_33_P3214466	MESP1	mesoderm posterior basic helix-loop-helix transcription factor 1	mesoderm posterior basic helix-loop-helix transcription factor 1 (MESP1)
A_23_P58763	PELO	pelota homolog (Drosophila)	pelota homolog (Drosophila) (PELO)
A_24_P88266	PROX1	prospero homeobox 1	prospero homeobox 1 (PROX1)
A_24_P157926	TNFAIP3	tumor necrosis factor, alpha-induced protein 3	tumor necrosis factor, alpha-induced protein 3 (TNFAIP3)

Supplemental Table 3. List of immortalized hepatocytes

Sample name	Introduced gene				Details
	TERT	***CDK4R24C***	***Cyclin D1***	***Tet-Off***	
Hep2004	+	+	+	+	*543:CSII-CMV-hTERT(MOI=3),1718:CSII-CMV-Tet-Off Advanced(MOI=3),1728:CSII-TRE-Tight-hCDK4R24C(MOI=3),1727:CSII-TRE-Tight-cyclin D1(MOI=3)*
Hep2013	+	+	+	+	*543:CSII-CMV-hTERT(MOI=3),1718:CSII-CMV-Tet-Off Advanced(MOI=3),1728:CSII-TRE-Tight-hCDK4R24C(MOI=3),1727:CSII-TRE-Tight-cyclin D1(MOI=3)*
Hep2017	+	+	+	-	*543:CSII-CMV-hTERT,1048:CSII-CMV-hCDK4R24C,1172:CSII-CMV-cyclin D1*
HepaMN (Hep2018)	+	+	+	+	*543:CSII-CMV-hTERT(MOI=3),1718:CSII-CMV-Tet-Off Advanced(MOI=5),1728:CSII-TRE-Tight-hCDK4R24C(MOI=10),1727:CSII-TRE-Tight-cyclin D1(MOI=10)*
Hep2020	+	+	+	+	*543:CSII-CMV-hTERT(MOI=3),1718:CSII-CMV-Tet-Off Advanced(MOI=5),1728:CSII-TRE-Tight-hCDK4R24C(MOI=10),1727:CSII-TRE-Tight-cyclin D1(MOI=10)*
Hep2040	+	+	+	-	*543:CSII-CMV-hTERT,1048:CSII-CMV-hCDK4R24C,1172:CSII-CMV-cyclin D1*
Hep2044	+	+	+	+	*543:CSII-CMV-hTERT,1718:CSII-CMV-Tet-Off Advanced,1728:CSII-TRE-Tight-hCDK4R24C,1727:CSII-TRE-Tight-cyclin D1*
Hep2045	+	+	+	+	*543:CSII-CMV-hTERT(MOI=3),1718:CSII-CMV-Tet-Off Advanced(MOI=5),1728:CSII-TRE-Tight-hCDK4R24C(MOI=10),1727:CSII-TRE-Tight-cyclin D1(MOI=10)*
Hep2022D	+	+	+	+	*543:CSII-CMV-hTERT(MOI=3),1718:CSII-CMV-Tet-Off Advanced(MOI=5),1728:CSII-TRE-Tight-hCDK4R24C(MOI=10),1727:CSII-TRE-Tight-cyclin D1(MOI=10)*
Hep2023D	+	+	+	-	*543:CSII-CMV-hTERT,1048:CSII-CMV-hCDK4R24C(MOI>10?),1172:CSII-CMV-cyclin D1(MOI>10?)*
Hep2039D	+	+	+	-	*543:CSII-CMV-hTERT(MOI-3),1048:CSII-CMV-hCDK4R24C(MOI=3),1172:CSII-CMV-cyclin D1(MOI=3)*

The contructions are detailed in Gene Therapy, 18:857, 2011.

Supplemental Table 4. List of liver samples

Sample name	Age	Sex	Recipient or Donor	Disease
Hep2001				MMA
Hep2002			R	CPS1D
Hep2003	14 years and 2 months	M	R	CAPV
Hep2004			R	BA
Hep2005R	7 months	F	R	BA
Hep2005D	30 years	F	D	Healthy liver
Hep2006	2 years and 8 months	F	R	BA
Hep2007	1 year and 1 month	F	R	BA
Hep2008	9 months	F	R	BA
Hep2009R	5 months	F	R	BA
Hep2009D	31 years	M	D	Healthy liver
Hep2010			R	OTCD
Hep2011	9 months	F	R	Alagile syndrome
Hep2012	8 months	F	R	BA
Hep2013	3 years and 10 months	F	R	BA
Hep2014	2 years and 1 month	F	R	Propionemia
Hep2015R	1 year and 1 month	F	R	BA
Hep2015D	40 years	M	D	Healthy liver
Hep2016	12 years and 5 months	F	R	BA
Hep2017	1 year and 3 months	F	R	GSDIb
Hep2018	4 years	F	R	BA
Hep2019	9 months	M	R	BA
Hep2020	6 years	M	R	CHF
Hep2021R	11 years and 5 months	M	R	GSDI
Hep2022D	26 years	M	D	Healthy liver
Hep2023D	32 years	M	D	Healthy liver
Hep2023R	10 months	F	R	BA
Hep2024D	21 years	F	D	Healthy liver
Hep2039D	36 years	F	D	Healthy liver
Hep2040	7 months	M	R	IHC
Hep2044	5 months	F	R	BA
Hep2045	5 years	M	R	BA

MMA: Methylmalonic acidemia
CPS1D: Carbamyl phosphate synthetase deficiency
CAPV: Congenital absence of the portal vein
BA: Biliary atresia
OTCD: Ornithine transcarbamylase deficiency
GSD: Glycogen storage disease
CHF: Congenital hepatic fibrosis
IHC: Intrahepatic cholestasis

Supplemental Table 5. Hepatic differentiation stage of hESCs used for principal component analysis (PCA)

Sample Number	Sample Name	Days cultivated after the start of the hepatic differentiation	Estimated Differentiation Stage	ESCs
1	SEES1-LGR5	0 days	Undifferentiated cells	SEES1
2	SEES4	0 days	Undifferentiated cells	SEES4
4	SEES5-P56	50 days	Mature hepatocyte-like cells	SEES5
5	LGR5F+I d7	7 days	Undifferentiated endodermal cells	SEES1
6	LGR5F+I d14	14 days	Immature hepatocyte-like cells	SEES1
7	LGR5F+H d7	7 days	Undifferentiated endodermal cells	SEES1
8	LGR5F+H d14	14 days	Immature hepatocyte-like cells	SEES1
9	LGR5H+I d7	7 days	Undifferentiated endodermal cells	SEES1
10	LGR5H+I d14	7 days	Undifferentiated endodermal cells	SEES1
11	LGR5XF- d7	7 days	Undifferentiated endodermal cells	SEES1
12	LGR5XF- d14	14 days	Immature hepatocyte-like cells	SEES1
13	LGR5XF32 d30	30 days	Mature hepatocyte-like cells	SEES1
14	LGR5XF32 d60	60 days	Mature hepatocyte-like cells	SEES1
15	SEES4-EB	14 days	Immature hepatocyte-like cells	SEES4

Human embryonic stem cell (hESC) lines SEES1, SEES4, and SEES5 were stably maintained in XF hESC culture medium containing 85% Knockout DMEM, 15% Knockout Serum Replacement XF CTS, 2 mM GlutaMAX-I, 0.1 mM NEAA, Pen-Strep, 50 µg/mL L-ascorbic acid 2-phosphate, 10 ng/mL heregulin-1β (recombinant human NRG-beta 1/HRG-beta 1 EGF domain), 200 ng/mL recombinant human IGF-1 (LONG R3-IGF-1; Sigma-Aldrich), and 20 ng/mL human bFGF (Akutsu et al., Regenerative Therapy; JCI insight). Undifferentiated hESCs were dissociated using dispase and plated on a dish coated with 0.1% human recombinant type I collagen peptide in 90 mm culture dishes. For hepatic differentiation, hESCs were cultured in XF hESC medium without growth factors (XF-KSR(-)) for 1 day and then in XF-KSR medium, which was replaced after 3 days with the XF hESC medium used as the differentiation medium. The differentiation medium was gently changed every 3–4 days until the indicated day.
